# Diphenyl pyridine intervention improves *S. aureus*-induced pneumonia by globally regulating transcriptome profile

**DOI:** 10.3389/fgene.2025.1624327

**Published:** 2025-09-10

**Authors:** Wei Duan, Qingfeng Zhu, Hai Ci, Jingang Zhang, Zhiwei Tian, Wanyu Li, Zhengfu Yang

**Affiliations:** ^1^ Clinical Laboratory, Shihezi University Affiliated Hospital of Traditional Chinese Medicine, Shihezi, Xinjiang, China; ^2^ Contagious Diseases Department, First Affiliated Hospital of Shihezi University, Shihezi, Xinjiang, China; ^3^ Department of Burn and Plastic Surgery, First Affiliated Hospital of Shihezi University, Shihezi, Xinjiang, China; ^4^ Laboratory Center, First Affiliated Hospital of Shihezi University, Shihezi, Xinjiang, China; ^5^ Respiratory and Critical Care Medicine Department, First Affiliated Hospital of Shihezi University, Shihezi, Xinjiang, China; ^6^ Laboratory Center, Shihezi University School of Medicine, Shihezi, Xinjiang, China

**Keywords:** DP, pneumonia, RNA-seq, hub genes, immune and inflammatory response

## Abstract

**Background:**

Pneumonia, induced by various bacteria or viruses, is a globally prevalent inflammatory disease that threatens the life of millions of people. *Staphylococcus aureus* (*S. aureus*) is a major pathogen of pneumonia and can be inhibited by Diphenyl pyrimidine (DP), while the underlying mechanisms are largely unknown.

**Methods:**

In this study, we conducted the *S. aureus*-induced rat pneumonia model and then performed DP treatment to inhibit the injury. Meanwhile, whole transcriptome sequencing (RNA-seq) experiment was performed to identify the dysregulated genes with expression and alternative splicing changes, as well as their enriched functions. Hub genes and immune cell proportion changes by DP were also identified to explore the underlying mechanism.

**Results:**

We identified 2,225 up and 1,257 down DEGs between DP and SA samples, and found they were significantly enriched in immune and inflammatory response pathways, as well as angiogenesis and apoptosis pathways. At the same time, DP treatment also significantly altered the alternative splicing profile, including 3898 AS genes and 416 co-regulated genes with DEGs. Functional analysis of co-regulated genes demonstrated they were enriched in immune response, signal transduction, and apoptosis regulation pathways. Finally, we identified ten hub genes by protein-protein network analysis from DEGs, including CCNA2, TOP2A, CDK1, ESPL1, KIF2C, PBK, UHRF1, RACGAP1, PCLAF, and RAD51 that were totally repressed by DP treatment.

**Conclusion:**

In summary, our study demonstrated that DP treatment can profoundly modulate the immune and inflammatory response by regulating the transcriptome profile of peripheral blood monocytes (PBMCs). The identified hub genes by DP treatment are potential therapeutic targets for *S. aureus*-induced pneumonia in future.

## Introduction

Pneumonia is a globally prevalent inflammatory disease with distinct seasonal and age distribution characteristics, particularly prevalent in immunocompromised populations such as the elderly and infants (Long et al., 2022). The main source of infection is pathogenic microorganisms, including various bacteria and viruses. Among the infected pathogens, *Staphylococcus aureus* (*S. aureus*) is a major pathogen responsible for severe and often life-threatening pneumonia, particularly in immunocompromised individuals, the elderly, and those with underlying respiratory conditions (He and Wunderink, 2020). *Staphylococcus aureus*-induced pneumonia is characterized by rapid disease progression, significant tissue damage, and high mortality rates, posing a substantial challenge to clinical management and public health. The pathogenesis of *S. aureus* pneumonia involves a complex interplay of bacterial virulence factors and host immune responses, leading to excessive inflammation, tissue injury, and impaired lung function (Cheung et al., 2021). Methicillin-resistant-*S.aureus* can induce the intestinal apoptosis, which is associated with the upregulation of apoptotic proteins Bid and Bax (Perrone et al., 2012). Previous study demonstrated that accumulation of γδ T cells in the lungs to *S. aureus* infection is beneficial for bacteria clearance and also contributes to the tissue damage (Cheng et al., 2012).

In recent years, the development of novel therapeutic strategies to combat *S. aureus* infections has become a critical area of research (Gopikrishnan et al., 2024). Traditional antibiotic treatments, while effective in many cases, face increasing challenges due to the emergence of antibiotic-resistant strains and the limitations of targeting specific bacterial pathogens (Ardal et al., 2020; Chinemerem Nwobodo et al., 2022). Therefore, there is a growing need for alternative approaches that can modulate the host immune response to mitigate the severity of the pneumonia induced by *S. aureus* and improve patient outcomes.

Based on our previous research and exploration, we have found that pyrimidine compounds have great potential in treating bacterial infectious diseases and can be used as research targets for antibacterial drugs (Trivedi et al., 2022), infective diseases, cancers, neurological disorders, and diabetes mellitus, and bacterial infections (Finger et al., 2023; Nammalwar and Bunce, 2024). Diphenyl pyridine (DP), a small molecule compound, has garnered attention for its potential immunomodulatory properties. Previous studies have suggested that DP can influence various cellular signaling pathways and gene expression profiles, thereby exerting anti-inflammatory and tissue-protective effects (Ghattas et al., 2017; Abdel-Maksoud et al., 2021). In our preliminary study, we used an animal model infected with *S. aureus* in rats and found that a class of compounds, Diphenyl pyrimidine (DP), had an effect on *S. aureus* suppression. The infection of *S. aureus* has a significant decrease after DP treatment, which may be achieved by targeting the NLRP3 protein (Duan et al., 2021). However, its role in the context of *S. aureus*-induced pneumonia has not been fully explored.

In this study, we aimed to investigate the therapeutic potential of DP in *S. aureus*-induced pneumonia by examining its effects on the global transcriptome profile of infected lung tissues. By elucidating the molecular mechanisms through which DP modulates the host response to *S. aureus* infection, we hope to provide new insights into the development of more effective therapeutic strategies for this devastating disease. Understanding the comprehensive changes in gene expression and the associated biological pathways regulated by diphenyl pyridine will not only enhance our knowledge of the host-pathogen interaction but also identify potential targets for future interventions.

## Materials and methods

### Reagents and bacterial strain

The 4,6-diphenylpyrimidine was purchased from GROSSERON (BD01107485), dissolved with DMSO when using. *Staphylococcus aureus* strain-8325-4 was obtained from the Wenzhou Kont Biology & Technology Co. Ltd. and cultured in tryptone soya broth.

### Animal studies

Female Sprague-Dawley rats (aged 6–8 weeks and weighing 180–220 g) were purchased from SPF (Suzhou) Biotechnology Co., Ltd. (License no. 202353764). All the rats were maintained in animal houses at 23 °C ± 2 °C, 45%–60% humidity and exposed to 12 h light/dark cycle. The rats after 1-week of acclimatization to the laboratory environment were separated into three groups of six each: *S. aureus* + PBS group (SA), *S. aureus* + diphenyl pyrimidine treatment groups (DP) and only PBS group as control (HL). This study was approved by the Biology Ethics Committee of Shihezi University with approval ID A2025-502.

### Infection and administration

The rats were anesthetized using ketamine (80 mg/kg) and xylazine (15 mg/kg) and then intraperitoneally inject with 100 μL suspension of *S. aureus* (4 × 10^8^ CFUs). The rats were intranasally administered 10 mg/kg doses of diphenylpyrimidine in physiological saline after 12 h of *S. aureus* infection according to our previous study (Duan et al., 2021). The mortality rate of rats was monitored during 72 h of the study.

### RNA extraction and sequencing (RNA-seq)

To explore the global immune or inflammatory response by DP treatment following previous study (Sadanandam et al., 2020), we used PBMCs as input from *S. aureus*-infected and DP treated rat samples. Total RNAs were extracted from peripheral blood using TRIzol Reagent (NO 15596026, Invitrogen) following the canonical RNA isolation method (Chomczynski and Sacchi, 1987). DNA was digested by DNaseI. RNA quality and integrity were determined by examining A260/A280 with NanodropTM OneCspectrophotometer (Thermo Fisher Scientific Inc.) and by 1.5% agarose gel electrophoresis, respectively. Then RNAs were quantified by Qubit3.0 with QubitTM RNA Broad Range Assay kit (Life Technologies Q10210). Total 2 μg RNAs were used for stranded RNA sequencing library preparation using KCTM Stranded mRNA Library Prep Kit for Illumina (DR08402, Seqhealth, China) following the manufacturer’s instruction. PCR products corresponding to 200-500 bps were enriched, quantified and finally sequenced on Novaseq 6000 sequencer (Illumina) with PE150 model.

### RNA-seq processing and alignment

Raw reads containing more than 2-N bases were first discarded. Then adaptors and low-quality bases were trimmed from raw sequencing reads using FASTX-Toolkit (Version 0.0.13). The short reads less than 16 nt were also dropped. After that, clean reads were aligned to the human genome by HISAT2 (Kim et al., 2019) allowing maximum four mismatches. Uniquely mapped reads were used for gene reads number counting and FPKM calculation (fragments per kilobase of transcript per million fragments mapped) (Trapnell et al., 2010).

### Differentially expressed genes (DEG) analysis

The R Bioconductor package DESeq2 (Love et al., 2014) was utilized to screen out the differentially expressed genes (DEGs). The corrected *P*-value by false discovery rate (FDR) method <0.05 and fold change >2 or <0.5 were set as the cut-off criteria for identifying DEGs.

### Alternative splicing analysis

The alternative splicing events (ASEs) and regulated alternative splicing events (RASEs) between the samples were defined and quantified by using the ABLas pipeline as described previously (Xia et al., 2017). In brief, ABLas detection of ten types of ASEs was based on the splice junction reads, including exon skipping (ES), alternative 5′splice site (A5SS), alternative 3’splice site (A3SS), mutually exclusive exons (MXE), mutually exclusive 5′UTRs (5pMXE), mutually exclusive 3′UTRs (3pMXE), A3SS&ES and A5SS&ES. The splicing ratio was calculated based on the constitutive splicing reads and alternative splicing reads for each ASEs. To assess regulated ASEs (RASEs), Student’s t-test was used to calculate the significance of the ratio alteration of ASEs. ASEs with significant *P*-value cutoff corresponding to a FDR of 5% were considered as RASEs.

### Estimation of immune cell fractions

We used CIBERSORT, a suite of machine learning tools designed for detecting the abundance of cell types in bulk RNA-seq dataset (Chen et al., 2018), to calculate the fractions of immune cell in this study. The default parameters were used for calculation.

### Protein-protein interaction (PPI) network construction and analysis

The STRING database was used to construct a PPI network for co-expressed genes. Then, the Cytoscape software (Shannon et al., 2003) was used to visualize the network interactions. The cytoHubba-MCC plug-in was used to explore important hub genes in an interactome network. Hub genes were defined as genes with top 10 ranked connectivity degrees.

### RT-qPCR experiment

We used reverse transcription and quantitative polymerase chain reaction (RT-qPCR) to validate the expression change of hub genes following previous study (Paizula et al., 2024). The Actb was used as the reference control. Bio-Rad S1000 with Hieff™ qPCR SYBR^®^ Green Master Mix (Low Rox Plus; YEASEN, China) was used for quantification. The 2^−ΔΔCT^ method (Livak and Schmittgen, 2001) was used for expression normalization. The primer sequences were presented in Supplementary Table S1.

### Statistical analysis

The comparison among different groups was analyzed by Student’s t-test for two groups and one-way ANOVA test for multiple groups. A difference with a *p*-value <0.01 or adjusted *p*-value <0.05 was considered statistically significant. The data were analyzed using GraphPad Prism 7 software.

## Results

### DP improves *Staphylococcus aureus*-induced pneumonia and has a wide range of targets

**FIGURE 1:**
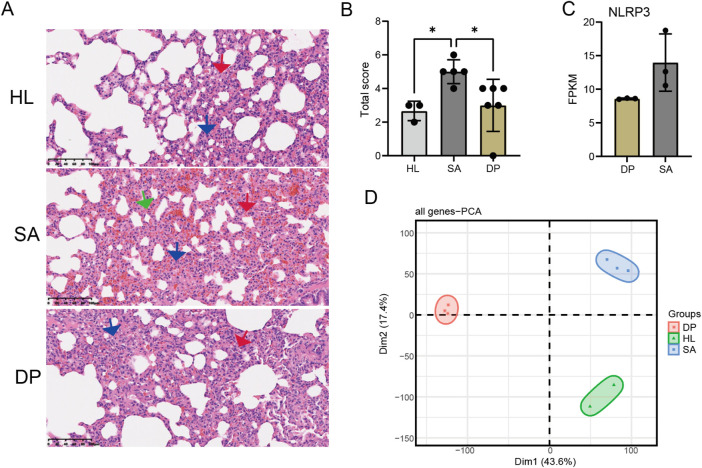
DP treatment protected *Staphylococcus aureus-*induced pneumonia and altered the transcriptome profile. **(A)** H&E staining of rat lung tissues showing the infiltration of inflammatory cells in three groups. **(B)** Bar plot showing the pathological scores in three groups. Outlier samples were discarded from each group. * *P*-value <0.05; One-way ANOVA test. **(C)** Bar plot showing the FPKM values of *Nlrp3* in SA and DP samples. **(D)** PCA result for all the detected genes in three groups.

Our previous study has demonstrated that DP can protect *S. aureus*-induced pneumonia probably by suppressing the expression of NLRP3 (Duan et al., 2021). However, the exact underlying mechanism is unknown. Then we performed a *S. aureus* infection (SA) rat model with DP treatment to investigate how DP alter the expression of genes. The results of H&E staining of rat lung tissues showed that *S. aureus* infection significantly increased the infiltration of inflammatory cells, while DP treatment suppressed the inflammatory infiltration (Figure 1A). At the same time, the pathological scoring of rat lung tissue showed that *S. aureus* infection had a higher score that the healthy control (HL) and treated groups (Figure 1B), indicating that DP treatment has a certain efficacy for *S. aureus* induced pneumonia. To better understand the pathogenesis of SA infection-induced pneumonia and the underlying mechanism of DP, we took peripheral blood of rats in three groups for transcriptome sequencing (RNA-seq). After obtaining the gene expression levels, principal component analysis (PCA) demonstrated a clear separation among these three groups (Figure 1C). Meanwhile, we also detected the downregulation of *Nlrp3* in DP treatment samples compared with SA samples (Figure 1D), consistent with our previous study. These results demonstrated that DP treatment can significantly alter the *S. aureus* induced pneumonia and the underlying transcriptome profile.

### DP treatment globally regulated the expression profile of genes associated with pneumonia

**FIGURE 2:**
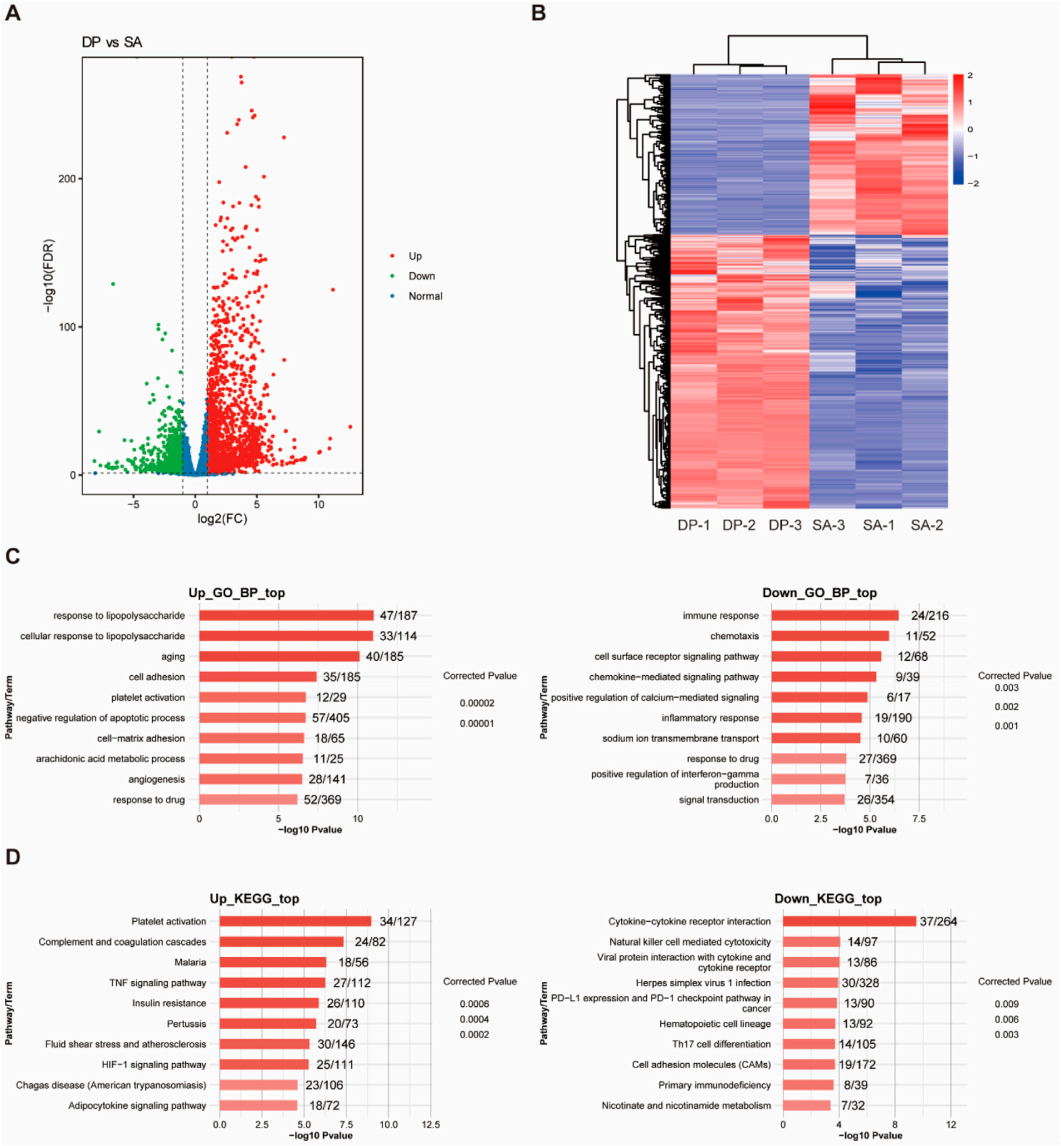
Identification and functional enrichment analysis of DEGs. **(A)** The volcano map of DEGs between DP and SA samples. **(B)** Heatmap of DEGs between DP and SA samples. **(C)** GO-BP enrichment analysis of upregulated and downregulated DEGs. **(D)** KEGG enrichment analysis of upregulated and downregulated DEGs.

We first identified the differentially expressed genes (DEGs) among the three groups. Compared with HL, *S. aureus* infection caused 567 upregulated and 193 downregulated DEGs (Supplementary Figure S1A). While DP treatment resulted in 2,225 upregulated and 1,257 downregulated DEGs (Figure 2A), indicating the global regulation on transcriptome of DP. Hierarchical clustering of the DEGs showed that they showed consistent expression pattern among the three replicates (Figure 2B; Supplementary Figure S1B). In summary, the number of genes induced by DP treatment far exceeded that of SA infection, suggesting that DP has a wide range of molecular targets.

To understand the functions of identified DEGs induced by the treatment of DP and *S. aureus*, we conducted GO and KEGG enrichment analysis. GO analysis results showed that DEGs regulated by SA infection were most enriched in inflammatory/immune response, cell proliferation, angiogenesis, and oxidative stress related biological process (Supplementary Figure S1C). DEGs regulated by DP treatment were most enriched in inflammatory/immune response, response to drug, and signal transduction related biological process (Figure 2C). KEGG analysis results showed that DEGs regulated by SA infection were enriched in Jak-STAT signaling, PI3K-Akt signaling pathway, RIG-I-like receptor signaling and metabolic pathways (Supplementary Figure S1D). DEGs regulated by DP treatment were enriched in HIF-1 signaling, TNF signaling, PD-1/PD-L1 pathway and Th17 cell differentiation (Figure 2D). In summary, SA infection leads to lung tissue damage through proinflammatory effects, imbalance of phagocytic function, and imbalance of metabolic and repair mechanisms. Meanwhile, DP treatment can protect the lung from SAP injury through the synergistic action of multiple mechanisms included anti-inflammatory, tissue repair and regeneration, immune and metabolic regulation.

### DP treatment globally changed the alternative splicing profile

**FIGURE 3:**
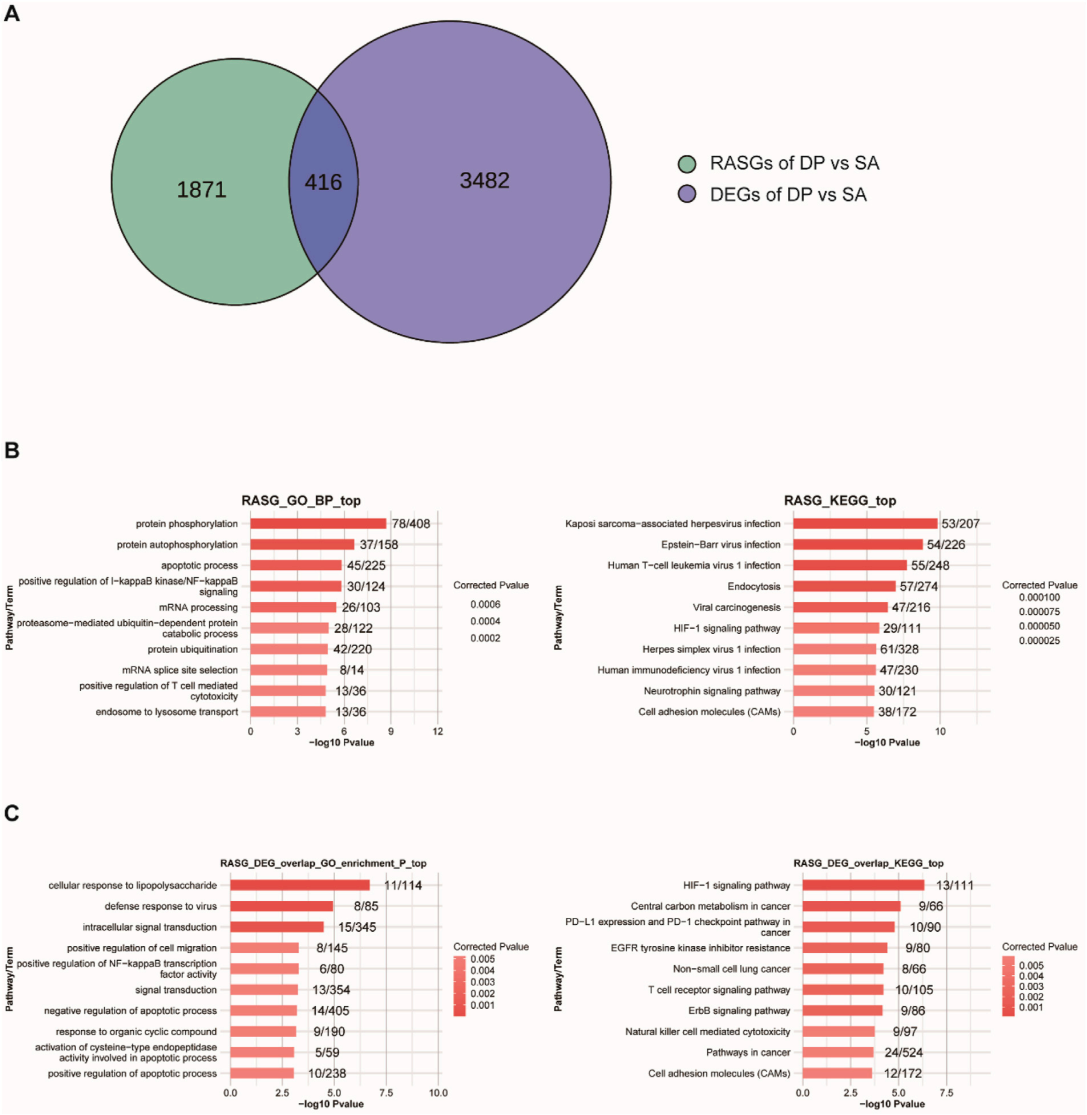
Genome-wide profiling of RASGs induced by DP. **(A)** Venn diagram showing the overlapped genes between RASGs and DEGs. **(B)** GO-BP and KEGG enrichment analysis of RASGs. **(C)** GO-BP and KEGG enrichment analysis of the overlapped genes between RASGs and DEGs.

Subsequently, we analyzed the change of alternative splicing based on the RNA-seq data, which can affect protein diversity and regulate gene expression, as well as involve in multiple diseases (Kim et al., 2018). Similar to the expression profiles of DEGs, DP treatment can also widely regulate alternative splicing events (ASEs) (Table 1). Specifically, the included AS events (Up) in DP vs. SA treatment were much more than the excluded events (Down), while the comparison between SA vs. control showed opposite trend (Table 1), indicating that DP treatment reversed the AS profile that was induced by SA infection. At the same time, we detected 416 overlapped genes between DEGs and DP-regulated alternative splicing genes (RASGs) (Figure 3A), suggesting that DP treatment can co-regulate the expression and AS profiles of hundreds of genes. Then, we performed enrichment analysis to annotate the functions of these RASGs by DP. The GO results demonstrated the significant enrichment of biological processes related to protein phosphorylation, apoptotic process, regulation of IKK/NF-κB signaling, mRNA processing, protein ubiquitination and regulation of T cell mediated cytotoxicity (Figure 3B, left panel). The KEGG results demonstrated significant enrichment of pathways related to virus infection, Endocytosis, HIF-1 signaling, Neurotrophin signaling, and Cell adhesion molecules (Figure 3B, right panel). We finally explored the functions of the 416 overlapping genes, and these genes were related to immune response, signal transduction, apoptosis regulation, cancer-related signaling pathways, cell metabolism, and adhesion (Figure 3C). In summary, these results demonstrated that DP treatment can also modulate the AS profile of genes that were tightly associated with immune response.

**TABLE 1:** Classification of RASEs between DP vs. SA and SA vs. HL.

Type	DP vs. SA		SA vs. HL	
	Up	Down	Up	Down
3pMXE	37	32	5	0
5pMXE	32	22	3	11
A3SS	471	130	40	52
A3SS&ES	23	16	1	4
A5SS	338	165	53	63
A5SS&ES	25	19	1	9
ES	156	99	24	24
IntronR	1,103	638	167	449
MXE	46	20	8	7
cassetteExon	158	98	15	30
Total	2,389	1,239	317	649

### Immune cell subpopulation analysis

**FIGURE 4:**
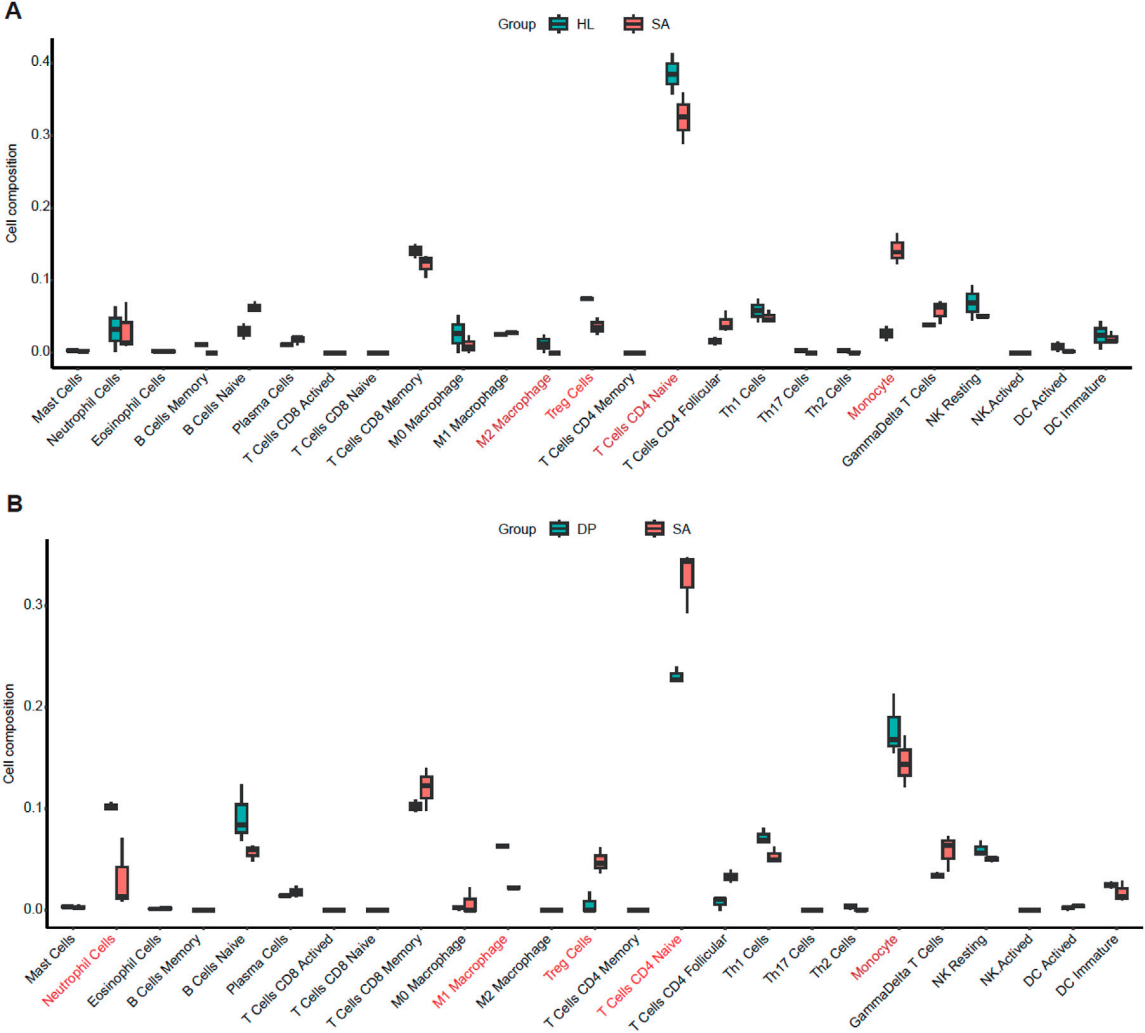
Immune cell subpopulation analysis based on CIBERSORT algorithm. **(A)** The proportion of immune cells between HL and SA groups. **(B)** The proportion of immune cells between DP and SA groups.

To investigate how DP treatment regulated the immune response, we evaluated the variations in immune cell subpopulation between different group using CIBERSORT algorithm based on the RNA-seq data of Peripheral blood. Compared with HL group, *S. aureus* infection decreased the proportion of M2 Macrophage, Treg Cells, T Cells CD4 Naive, Th1 Cells, and increased the proportion of B Cells Naive, T Cells CD4 Follicular, Monocyte, γδ T cells (Figure 4A). Compared with SA group, DP intervention increased the proportion of neutrophils, M1 macrophages, B Cells Naive, Th1 Cells and Monocyte, and decreased T Cells CD8 Memory, Treg Cells, T Cells CD4 naive. The proportion of γδ T cells was also obviously changed (Figure 4B). Specifically, the neutrophil cells, M1 macrophages, and monocytes were significantly increased with high composition in DP samples (Figure 4B). Overall, DP regulates the proportion and function of immune cell subsets in multiple dimensions to avoid immunopathological damage while removing *S. aureus*.

### PPI network construction and hub gene identification

**FIGURE 5:**
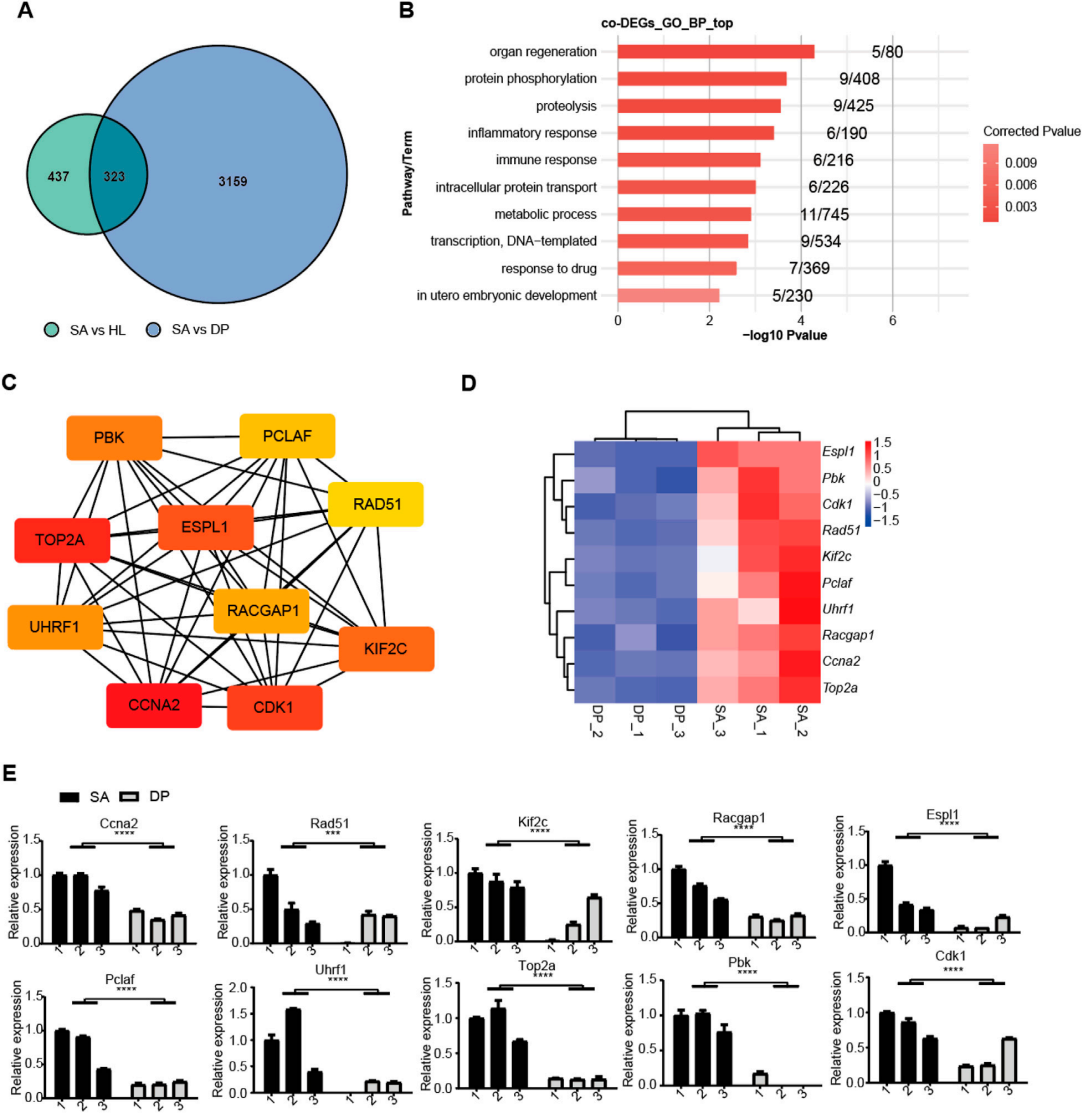
Selection and analysis of hub genes that were co-regulated by SA and DP. **(A)** Venn diagram shows the comparison of deregulated genes between SA and DP. **(B)** GO-BP enrichment analysis of co-DEGs. **(C)** Hub genes in the PPI-network identified using cytoHubba-MCC. **(D)** Heatmap showing the expression levels of hub genes between SA and DP. **(E)** Bar plot showing the decreased expression levels of these ten hub genes in DP samples. N = 3; One-way ANOVA test, *** *P*-value <0.001; **** *P*-value <0.0001.

To further identify the key regulators (hub genes) that were induced by DP and SA treatment, we performed a protein-protein interaction (PPI) network analysis. First, we identified 323 Co-regulated Genes (co-DEGs) between DP vs. SA group and SA vs. HL group (Figure 5A) and used them for following analysis. GO-BP enrichment analysis revealed that these co-DEGs were mainly enriched in organ regeneration, inflammatory/immune response, metabolic process and other pathways (Figure 5B). The interaction network between proteins encoded by these co-DEGs was constructed using the STRING database. After visualization by Cytoscape software, we identified hub genes using cytoHubba-MCC plugin. The results showed that CCNA2, TOP2A, CDK1, ESPL1, KIF2C, PBK, UHRF1, RACGAP1, PCLAF, and RAD51 were the top 10 hub genes (Figure 5C). Most of these hub genes play a key role in cell proliferation and are associated with the occurrence and development of many diseases, especially cancer (Supplementary Table S2). Interestingly, all these ten hub genes had similar expression patterns and were inhibited by DP treatment (Figure 5D), indicating that these genes were deeply involved in the DP protection of SA-induced Pneumonia. To confirm the expression pattern of these ten hub genes, we performed RT-qPCR experiment and found all of these hub genes were downregulated in DP samples, although there existed individual variation (Figure 5E).

## Discussion

Pneumonia remains a significant global health concern, particularly due to its high morbidity and mortality rates (Niederman and Torres, 2022). Among the various pathogens responsible for pneumonia, *Staphylococcus aureus* (*S. aureus*) stands out as a major pathogen, often leading to severe and life-threatening infections (Bai et al., 2022). In this study, we systematically investigated the therapeutic potential and underlying mechanism of Diphenyl pyrimidine (DP) in mitigating *S. aureus*-induced pneumonia by constructing rat pneumonia model and performing RNA-seq analysis using PBMC samples. Our results demonstrated that DP treatment profoundly altered the transcriptome profile, including gene expression and alternative splicing. The dysregulated genes, including 10 hub genes identified by PPI network, were significantly enriched in immune and inflammatory associated pathways, suggesting that DP treatment can alter the immune system to protect hosts from *S. aureus*-induced lung injury and pneumonia. In summary, our results provide novel insights into the underlying mechanisms of DP in pneumonia treatment through comprehensive transcriptome analysis and experimental validation.

### Mechanisms of DP in inhibiting *Staphylococcus aureus*-Induced pneumonia

Previous study has demonstrated that complex bacterial toxins, including *S. aureus* infection, can be targeted *in vivo* by drug-like small molecules, which also inhibit the lung injury of hosts (Shekhar et al., 2025), indicating the potential application of small molecules in pneumonia treatment. Based on our previous research foundation, we identified a substantial number of differentially expressed genes (DEGs) between DP-treated and *S. aureus*-infected samples, including 2,225 upregulated and 1,257 downregulated DEGs. These DEGs were significantly enriched in pathways related to immune and inflammatory responses, as well as angiogenesis and apoptosis pathways that were associated with pneumonia pathogenesis (Zheng et al., 2021). Very few studies have reported the functions of DP in disease treatment. One abstract reported that Diphenyl-pyridines can be promising scaffolds as antiviral, anticancer and theranostic agents (Cianciusi, 2023). Another study demonstrated that the pyrimidine derivatives showed anti-microbial, anticancer, anti-inflammatory, anti-tubercular, anti-convulsant, antihypertensive, anthelmintic, antidepressant, analgesic, and anti-hyperglycemic activities (Natarajan et al., 2022). In this study, the expression levels of immune function genes that involve in pneumonia pathogenesis (Zhu et al., 2023), was significantly regulated by DP treatment. This enrichment suggests that DP may exert its therapeutic effects by modulating these critical biological processes. For instance, the upregulation of genes involved in immune response pathways can enhance the host’s ability to combat the infection, while the regulation of apoptosis-related genes might help in reducing tissue damage and promoting recovery (Kazzaz et al., 2000). Meanwhile, how DP treatment alters the expression pattern of these genes is unknown, implying that further studies are necessary to deeper identify the underlying regulatory mechanism.

At the same time, we detected the significantly proportional change of immune cell types by CIBERSORT algorithm analysis, including neutrophil cells, M1 macrophages, and monocytes. It has been reported that neutrophil can be recruited to the lungs during Bacterial Pneumonia and induce the initial innate immune response to defense the bacteria, including *S. aureus* (Craig et al., 2009). M1 macrophages were increased by DP treatment but not for M2 macrophage. One previous study demonstrated that *Pneumocystis* pneumonia can induce an M1 response both *in vitro* and *in vivo*, and treatment with both M1 and M2 cells significantly improved survival of *P. carinii*-infected immunosuppressed hosts (Nandakumar et al., 2017), indicating that DP may inhibit *S. aureus* infection through M1 macrophage activation. Monocytes, one type of mononuclear phagocytes, play critical role in antimicrobial defenses probably through secreting multiple kinds of cytokine and chemokine receptors (Seo et al., 2011; Chen et al., 2013). The Treg cells were significantly decreased in DP treatment samples. One study reported that the Th17/Treg ratio was higher in patients with refractory *Mycoplasma* pneumoniae (MP) pneumonia than that with macrolide responsive MP pneumonia (Guo et al., 2016). In summary, these results indicate that DP can contribute to the treatment of pneumonia probably through regulating the activation or recruitment multiple kinds of immune cells, which needs to be further validated using advanced technologies in future.

### Alternative splicing and co-regulated genes

In addition to changes in gene expression, DP treatment also significantly altered the alternative splicing (AS) profile, with a substantial number of alternatively spliced genes and co-regulated genes with DEGs were identified. AS plays an essential role in protein diversity of eukaryotic organisms and the development of many diseases (Montes et al., 2019; Jiang and Chen, 2021). DP treatment modulated the pattern of thousands of AS events and showed a reverse pattern of *S. aureus*-induced AS pattern, suggesting that DP may also inhibit the *S. aureus*-induced injury by changing the AS profile. It has been reported that *S. aureus* infection can induce the global AS profile change in mammary gland tissues of cows, including a lot of immune-related genes (Wang et al., 2016). Similar results were also observed in *S. aureus*-induced mastitis of mice, with 30% more splice variants than the reference genome (Mitra et al., 2020). Interestingly, we observed 416 overlapped genes between DEGs and RASGs, further implying that alternative splicing is also a crucial mechanism for generating protein diversity and regulating gene expression (Ule and Blencowe, 2019). Functional analysis revealed that these co-regulated genes were also enriched in pathways associated with immune response, signal transduction, and apoptosis regulation. These results suggest that the changes in AS profile observed in our study may contribute to the fine-tuning of the immune and inflammatory responses, further highlighting the multifaceted effects of DP on the transcriptome profile.

### Identification of hub genes

Through protein-protein interaction network analysis, we identified ten hub genes that all were repressed by DP treatment, including CCNA2, TOP2A, CDK1, ESPL1, KIF2C, PBK, UHRF1, RACGAP1, PCLAF, and RAD51. The downregulation of these hub genes by DP suggests their potential roles in mediating the inhibitory effects of DP on *S. aureus*-induced pneumonia. For example, CCNA2 and CDK1 are key regulators of cell cycle progression, and their repression may lead to cell cycle arrest (Enserink and Chymkowitch, 2022; Zhou et al., 2024), thereby reducing the proliferation of infected cells and limiting the spread of the infection. TOP2A is an important regulator in transcription and cancer by regulating chromatin topology and maintaining genomic integrity (Uuskula-Reimand and Wilson, 2022). The downregulation of apoptosis-related genes such as TOP2A and RAD51 may promote cell survival and tissue repair (Im et al., 2018), contributing to the recovery from pneumonia. KIF2C and PCLAF have been identified as critical genes in COVID-19 and may be potential biomarkers and treatment targets (Li et al., 2022; Zhang et al., 2022). UHRF1 is a DNA methylation maintenance protein that can promote regulatory T cell-mediated recovery following viral pneumonia (Joudi et al., 2025), suggesting its potential role in DP-mediated pneumonia recovery. Although these hub genes are rarely investigated in *S. aureus*-induced pneumonia, we propose that they can serve as potential regulatory molecules in the therapy of pneumonia that are modulated by DP treatment. Further validation of these hub genes using Western blot and other methods can support their dysregulation by DP treatment and underscores their potential as therapeutic targets. Meanwhile, it is also important to explore the expression pattern of these target genes in the affected lung tissue to further validate the discovery in PBMCs.

In summary, the findings of our study provide valuable insights into the mechanisms underlying the inhibitory effects of DP on *S. aureus*-induced pneumonia. The identified hub genes regulated by DP treatment offer potential therapeutic targets for the treatment of this disease. Future research could focus on exploring the specific roles of these hub genes in the pathogenesis of *S. aureus*-induced pneumonia and developing targeted therapies based on these findings. Additionally, further studies on the effects of DP on other aspects of the immune response, such as cytokine production and immune cell activation, could provide a more comprehensive understanding of its therapeutic potential. Other experiments focusing on the clinical translation of DP, such as delivery method, safety in human beings, and effects of off-target, should also be seriously considered before it can be used in pneumonia treatment. Meanwhile, earlier or repeated dosing of DP treatment can also be considered in further research studies. In conclusion, our study demonstrates that DP treatment can profoundly modulate the immune and inflammatory response by regulating the transcriptome profile of PBMCs. The identified hub genes and pathways regulated by DP treatment provide a foundation for future research and the development of novel therapeutic strategies for *S. aureus*-induced pneumonia.

## Data Availability

The datasets presented in this study can be found in online repositories. The names of the repository/repositories and accession number(s) can be found in the article/Supplementary Material.

## References

[B1] Abdel-Maksoud M. S. Mohamed Hassan R. Abdel-Sattar El-Azzouny A. Nabil Aboul-Enein M. Oh C. H. (2021). Anticancer profile and anti-inflammatory effect of new N-(2-4-1,3-diphenyl-1H-pyrazol-4-yl)pyridine sulfonamide derivatives. Bioorg Chem. 117, 105424. 10.1016/j.bioorg.2021.105424 34678604

[B2] Ardal C. Balasegaram M. Laxminarayan R. Mcadams D. Outterson K. Rex J. H. (2020). Antibiotic development - economic, regulatory and societal challenges. Nat. Rev. Microbiol. 18, 267–274. 10.1038/s41579-019-0293-3 31745330

[B3] Bai A. D. Lo C. K. L. Komorowski A. S. Suresh M. Guo K. Garg A. (2022). *Staphylococcus aureus* bacteraemia mortality: a systematic review and meta-analysis. Clin. Microbiol. Infect. 28, 1076–1084. 10.1016/j.cmi.2022.03.015 35339678

[B4] Chen L. Zhang Z. Barletta K. E. Burdick M. D. Mehrad B. (2013). Heterogeneity of lung mononuclear phagocytes during pneumonia: contribution of chemokine receptors. Am. J. Physiol. Lung Cell Mol. Physiol. 305, L702–L711. 10.1152/ajplung.00194.2013 24056971 PMC3840272

[B5] Chen B. Khodadoust M. S. Liu C. L. Newman A. M. Alizadeh A. A. (2018). Profiling Tumor infiltrating immune cells with CIBERSORT. Methods Mol. Biol. 1711, 243–259. 10.1007/978-1-4939-7493-1_12 29344893 PMC5895181

[B6] Cheng P. Liu T. Zhou W. Y. Zhuang Y. Peng L. S. Zhang J. Y. (2012). Role of gamma-delta T cells in host response against Staphylococcus aureus-induced pneumonia. BMC Immunol. 13, 38. 10.1186/1471-2172-13-38 22776294 PMC3524664

[B7] Cheung G. Y. C. Bae J. S. Otto M. (2021). Pathogenicity and virulence of *Staphylococcus aureus* . Virulence 12, 547–569. 10.1080/21505594.2021.1878688 33522395 PMC7872022

[B8] Chinemerem Nwobodo D. Ugwu M. C. Oliseloke Anie C. Al-Ouqaili M. T. S. Chinedu Ikem J. Victor Chigozie U. (2022). Antibiotic resistance: the challenges and some emerging strategies for tackling a global menace. J. Clin. Lab. Anal. 36, e24655. 10.1002/jcla.24655 35949048 PMC9459344

[B9] Chomczynski P. Sacchi N. (1987). Single-step method of RNA isolation by acid guanidinium thiocyanate-phenol-chloroform extraction. Anal. Biochem. 162, 156–159. 10.1006/abio.1987.9999 2440339

[B10] Cianciusi A. (2023). 4, 6-Diphenyl-pyridines/pyrimidines and pyrazolo [3, 4-d] pyrimidines: promising scaffolds as antiviral, anticancer and Theranostic agents.

[B11] Craig A. Mai J. Cai S. Jeyaseelan S. (2009). Neutrophil recruitment to the lungs during bacterial pneumonia. Infect. Immun. 77, 568–575. 10.1128/IAI.00832-08 19015252 PMC2632043

[B12] Duan W. Qin F. Wu D. Dai Y. (2021). Diphenyl pyrimidine exhibits protective effect on *Staphylococcus aureus* pneumonia in rat model by targeting NLRP3 expression. Microb. Pathog. 161, 105168. 10.1016/j.micpath.2021.105168 34478857

[B13] Enserink J. M. Chymkowitch P. (2022). Cell cycle-dependent transcription: the cyclin dependent kinase Cdk1 is a direct regulator of basal transcription machineries. Int. J. Mol. Sci. 23, 1293. 10.3390/ijms23031293 35163213 PMC8835803

[B14] Finger V. Kufa M. Soukup O. Castagnolo D. Roh J. Korabecny J. (2023). Pyrimidine derivatives with antitubercular activity. Eur. J. Med. Chem. 246, 114946. 10.1016/j.ejmech.2022.114946 36459759

[B15] Ghattas A.-E.-B. A. Khodairy A. Moustafa H. M. Hussein B. R. Farghaly M. M. Aboelez M. O. (2017). Synthesis, *in vitro* antibacterial and *in vivo* anti-inflammatory activity of some new pyridines. Pharm. Chem. J. 51, 652–660. 10.1007/s11094-017-1670-8

[B16] Gopikrishnan M. Haryini S. C G. P. D. (2024). Emerging strategies and therapeutic innovations for combating drug resistance in *Staphylococcus aureus* strains: a comprehensive review. J. Basic Microbiol. 64, e2300579. 10.1002/jobm.202300579 38308076

[B17] Guo H. He Z. Li M. Wang T. Zhang L. (2016). Imbalance of peripheral blood Th17 and treg responses in children with refractory Mycoplasma pneumoniae pneumonia. J. Infect. Chemother. 22, 162–166. 10.1016/j.jiac.2015.12.006 26806148

[B18] He H. Wunderink R. G. (2020). *Staphylococcus aureus* pneumonia in the community. Semin. Respir. Crit. Care Med. 41, 470–479. 10.1055/s-0040-1709992 32521547

[B19] Im J. Lawrence J. Seelig D. Nho R. S. (2018). FoxM1-dependent RAD51 and BRCA2 signaling protects idiopathic pulmonary fibrosis fibroblasts from radiation-induced cell death. Cell Death Dis. 9, 584. 10.1038/s41419-018-0652-4 29789556 PMC5964221

[B20] Jiang W. Chen L. (2021). Alternative splicing: human disease and quantitative analysis from high-throughput sequencing. Comput. Struct. Biotechnol. J. 19, 183–195. 10.1016/j.csbj.2020.12.009 33425250 PMC7772363

[B21] Joudi A. M. Gurkan J. K. Liu Q. Acosta M. a.T. Helmin K. A. Morales-Nebreda L. (2025). Maintenance DNA methylation is required for induced regulatory T cell reparative function following viral pneumonia. bioRxiv 2025, 2025.02.25.640199. 10.1101/2025.02.25.640199 40956625 PMC12618063

[B22] Kazzaz J. A. Horowitz S. Xu J. Khullar P. Niederman M. S. Fein A. M. (2000). Differential patterns of apoptosis in resolving and nonresolving bacterial pneumonia. Am. J. Respir. Crit. Care Med. 161, 2043–2050. 10.1164/ajrccm.161.6.9806158 10852786

[B23] Kim H. K. Pham M. H. C. Ko K. S. Rhee B. D. Han J. (2018). Alternative splicing isoforms in health and disease. Pflugers Arch. 470, 995–1016. 10.1007/s00424-018-2136-x 29536164

[B24] Kim D. Paggi J. M. Park C. Bennett C. Salzberg S. L. (2019). Graph-based genome alignment and genotyping with HISAT2 and HISAT-Genotype. Nat. Biotechnol. 37, 907–915. 10.1038/s41587-019-0201-4 31375807 PMC7605509

[B25] Li X. Zhou X. Ding S. Chen L. Feng K. Li H. (2022). Identification of transcriptome biomarkers for severe COVID-19 with machine learning methods. Biomolecules 12, 1735. 10.3390/biom12121735 36551164 PMC9775121

[B26] Livak K. J. Schmittgen T. D. (2001). Analysis of relative gene expression data using real-time quantitative PCR and the 2(-Delta Delta C(T)) method. Methods 25, 402–408. 10.1006/meth.2001.1262 11846609

[B27] Long M. E. Mallampalli R. K. Horowitz J. C. (2022). Pathogenesis of pneumonia and acute lung injury. Clin. Sci. (Lond) 136, 747–769. 10.1042/CS20210879 35621124 PMC9429452

[B28] Love M. I. Huber W. Anders S. (2014). Moderated estimation of fold change and dispersion for RNA-Seq data with DESeq2. Genome Biol. 15, 550. 10.1186/s13059-014-0550-8 25516281 PMC4302049

[B29] Mitra S. D. Ganaie F. Bankar K. Velu D. Mani B. Vasudevan M. (2020). Genome-wide analysis of mammary gland shows modulation of transcriptome landscape with alternative splice variants in *Staphylococcus aureus* mastitis in mice. Gene 735, 144278. 10.1016/j.gene.2019.144278 31821873

[B30] Montes M. Sanford B. L. Comiskey D. F. Chandler D. S. (2019). RNA splicing and disease: animal models to therapies. Trends Genet. 35, 68–87. 10.1016/j.tig.2018.10.002 30466729 PMC6339821

[B31] Nammalwar B. Bunce R. A. (2024). Recent advances in pyrimidine-based drugs. Pharm. (Basel) 17, 104. 10.3390/ph17010104 38256937 PMC10820437

[B32] Nandakumar V. Hebrink D. Jenson P. Kottom T. Limper A. H. (2017). Differential macrophage polarization from pneumocystis in immunocompetent and immunosuppressed hosts: potential adjunctive therapy during pneumonia. Infect. Immun. 85, e00939-16. 10.1128/IAI.00939-16 27993972 PMC5328482

[B33] Natarajan R. Anthoni Samy H. N. Sivaperuman A. Subramani A. (2022). Structure-activity relationships of pyrimidine derivatives and their biological activity - a review. Med. Chem. 19, 10–30. 10.2174/1573406418666220509100356 35579151

[B34] Niederman M. S. Torres A. (2022). Severe community-acquired pneumonia. Eur. Respir. Rev. 31, 220123. 10.1183/16000617.0123-2022 36517046 PMC9879347

[B35] Paizula X. Wulaying A. Chen D. Ou J. (2024). KHSRP has oncogenic functions and regulates the expression and alternative splicing of DNA repair genes in breast cancer MDA-MB-231 cells. Sci. Rep. 14, 14694. 10.1038/s41598-024-64687-0 38926398 PMC11208542

[B36] Perrone E. E. Jung E. Breed E. Dominguez J. A. Liang Z. Clark A. T. (2012). Mechanisms of methicillin-resistant *Staphylococcus aureus* pneumonia-induced intestinal epithelial apoptosis. Shock 38, 68–75. 10.1097/SHK.0b013e318259abdb 22592747 PMC3392021

[B37] Sadanandam A. Bopp T. Dixit S. Knapp D. Emperumal C. P. Vergidis P. (2020). A blood transcriptome-based analysis of disease progression, immune regulation, and symptoms in coronavirus-infected patients. Cell Death Discov. 6, 141. 10.1038/s41420-020-00376-x 33293514 PMC7721861

[B38] Seo S. U. Kwon H. J. Ko H. J. Byun Y. H. Seong B. L. Uematsu S. (2011). Type I interferon signaling regulates Ly6C(hi) monocytes and neutrophils during acute viral pneumonia in mice. PLoS Pathog. 7, e1001304. 10.1371/journal.ppat.1001304 21383977 PMC3044702

[B39] Shannon P. Markiel A. Ozier O. Baliga N. S. Wang J. T. Ramage D. (2003). Cytoscape: a software environment for integrated models of biomolecular interaction networks. Genome Res. 13, 2498–2504. 10.1101/gr.1239303 14597658 PMC403769

[B40] Shekhar A. Di Lucrezia R. Jerye K. Korotkov V. S. Harmrolfs K. Rox K. (2025). Highly potent quinoxalinediones inhibit alpha-hemolysin and ameliorate *Staphylococcus aureus* lung infections. Cell Host Microbe 33, 560–572.e21. 10.1016/j.chom.2025.03.006 40168998

[B41] Trapnell C. Williams B. A. Pertea G. Mortazavi A. Kwan G. Van Baren M. J. (2010). Transcript assembly and quantification by RNA-seq reveals unannotated transcripts and isoform switching during cell differentiation. Nat. Biotechnol. 28, 511–515. 10.1038/nbt.1621 20436464 PMC3146043

[B42] Trivedi H. D. Joshi V. B. Patel B. Y. (2022). Pyrazole bearing pyrimidine analogues as the privileged scaffolds in antimicrobial drug discovery: a review. Anal. Chem. Lett. 12, 147–173. 10.1080/22297928.2021.1910565

[B43] Ule J. Blencowe B. J. (2019). Alternative splicing regulatory networks: functions, mechanisms, and evolution. Mol. Cell 76, 329–345. 10.1016/j.molcel.2019.09.017 31626751

[B44] Uuskula-Reimand L. Wilson M. D. (2022). Untangling the roles of TOP2A and TOP2B in transcription and cancer. Sci. Adv. 8, eadd4920. 10.1126/sciadv.add4920 36322662 PMC9629710

[B45] Wang X. G. Ju Z. H. Hou M. H. Jiang Q. Yang C. H. Zhang Y. (2016). Deciphering transcriptome and complex alternative splicing transcripts in mammary gland tissues from cows naturally infected with *Staphylococcus aureus* mastitis. PLoS One 11, e0159719. 10.1371/journal.pone.0159719 27459697 PMC4961362

[B46] Xia H. Chen D. Wu Q. Wu G. Zhou Y. Zhang Y. (2017). CELF1 preferentially binds to exon-intron boundary and regulates alternative splicing in HeLa cells. Biochim. Biophys. Acta 1860, 911–921. 10.1016/j.bbagrm.2017.07.004 28733224

[B47] Zhang F. Yu C. Xu W. Li X. Feng J. Shi H. (2022). Identification of critical genes and molecular pathways in COVID-19 myocarditis and constructing gene regulatory networks by bioinformatic analysis. PLoS One 17, e0269386. 10.1371/journal.pone.0269386 35749386 PMC9231758

[B48] Zheng D. J. Abou Taka M. Heit B. (2021). Role of apoptotic cell clearance in pneumonia and inflammatory lung disease. Pathogens 10, 134. 10.3390/pathogens10020134 33572846 PMC7912081

[B49] Zhou H. Y. Wang Y. C. Wang T. Wu W. Cao Y. Y. Zhang B. C. (2024). CCNA2 and NEK2 regulate glioblastoma progression by targeting the cell cycle. Oncol. Lett. 27, 206. 10.3892/ol.2024.14339 38516683 PMC10956385

[B50] Zhu Y. Luo Y. Li L. Jiang X. Du Y. Wang J. (2023). Immune response plays a role in Mycoplasma pneumoniae pneumonia. Front. Immunol. 14, 1189647. 10.3389/fimmu.2023.1189647 37304280 PMC10250694

