# Supplementary material for: Diphenyl pyridine intervention improves S. aureus-induced pneumonia by globally regulating transcriptome profile
Source: Front Genet. 2025 Sep 10;16:1624327. doi: 10.3389/fgene.2025.1624327 (PMC12457106; doi:10.3389/fgene.2025.1624327)
Supplement: Supplementary file 1 [file Supplementaryfile1.docx]

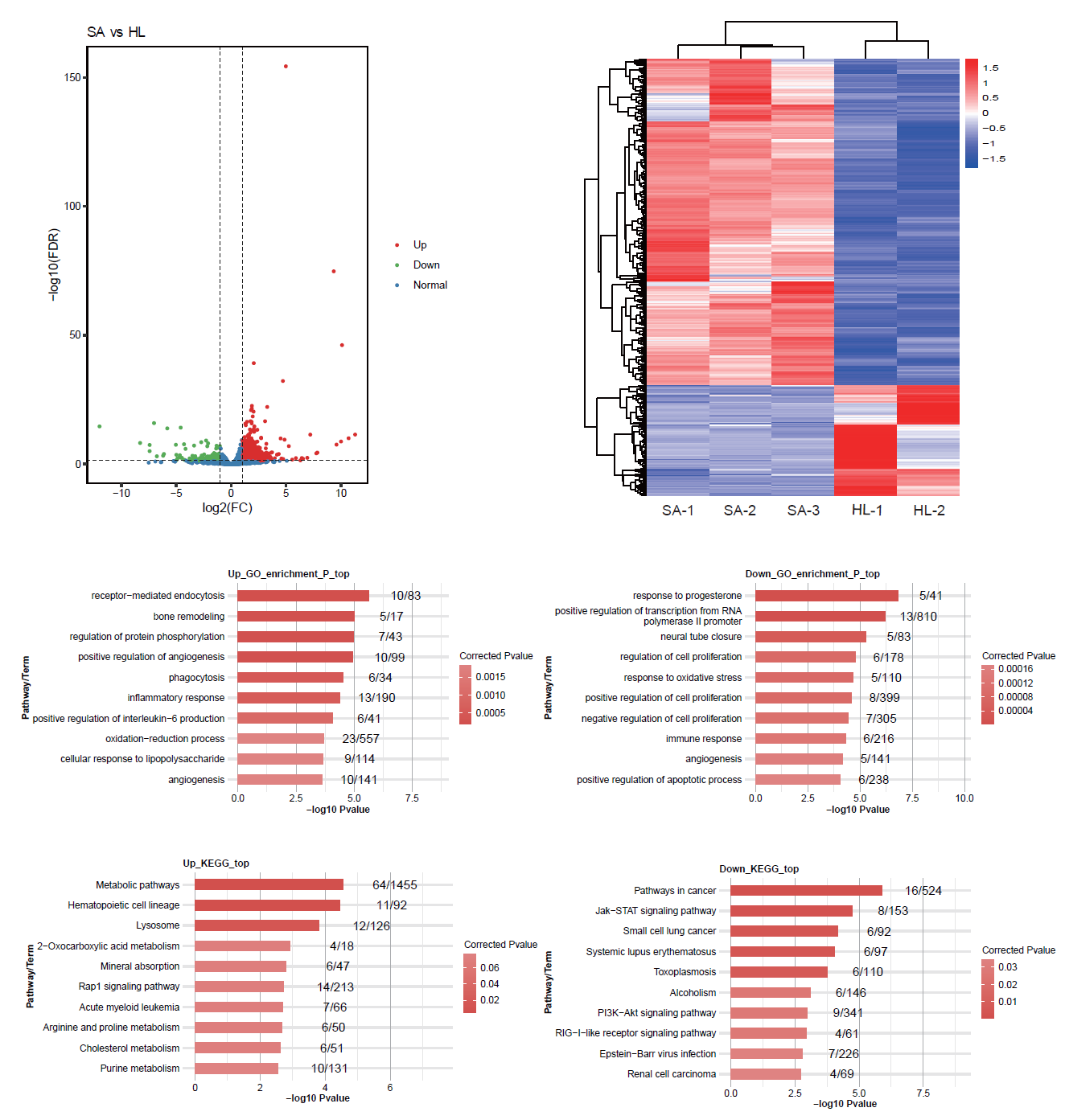


**Figure S1. Identification and functional enrichment analysis of DEGs.**

1. The volcano map of DEGs between SA and HL samples.
2. Heatmap of DEGs between SA and HL samples.
3. GO-BP enrichment analysis of up-regulated and down-regulated DEGs.
4. KEGG enrichment analysis of up-regulated and down-regulated DEGs.
