# Supplementary material for: Diphenyl pyridine intervention improves S. aureus-induced pneumonia by globally regulating transcriptome profile
Source: Front Genet. 2025 Sep 10;16:1624327. doi: 10.3389/fgene.2025.1624327 (PMC12457106; doi:10.3389/fgene.2025.1624327)
Supplement: Supplementary file 2 [file Table1.docx]

Table S1. The primer sequences for RT-qPCR experiment in this study.

| Ccna2-RAT-F: | CAGTGTTTCTCTTCTCAACCC |
| --- | --- |
| Ccna2-RAT-R: | TGTCTGTGGCTATACCATCTAC |
| Rad51-F: | TCTGTAAGTGGGAATGGGTGTT |
| Rad51-R: | AACTCGCTTAGGTGAAGTCTCA |
| Kif2c-F: | TGACTTGGAGACCTTCGTGAA |
| Kif2c-R: | GGTGCCGTTTCTTGCTGTT |
| Racgap1-F: | TTTGGGAACAAGAGCAAGTCTG |
| Racgap1-R: | TGAGGAGCGAACAGGTGTG |
| Espl1-F: | TGTGACTGACCGTGATATTGAC |
| Espl1-R: | GGCTTGGCTGACATAGTGGA |
| Pclaf-F: | TGGAGGGAACCCAGTCTGT |
| Pclaf-R: | CCACTGCTTCCTGCTTCTTC |
| Uhrf1-F: | AGATCTTCCTGAGCAAGGTGAA |
| Uhrf1-R: | CTTACAAACGTTGTGCTGACAC |
| Top2a-F: | GGCATTGTCGTCTTCTCTGATT |
| Top2a-R: | GGATAGGAAAGGAGGCAGGAA |
| Pbk-F: | ATGGCATTATTACTGACAAGGC |
| Pbk-R: | TCATCATCATCATCTGGAAGGT |
| Cdk1-F: | GCACCCATACTTTGACGACTT |
| Cdk1-R: | CAAGACAGGAAGAGCCAACAG |
| Actin-F: | CAGGGTGTGATGGTGGGTATGG |
| Actin-R: | AGTTGGTGACAATGCCGTGTTC |
