# Supplementary material for: Diphenyl pyridine intervention improves S. aureus-induced pneumonia by globally regulating transcriptome profile
Source: Front Genet. 2025 Sep 10;16:1624327. doi: 10.3389/fgene.2025.1624327 (PMC12457106; doi:10.3389/fgene.2025.1624327)
Supplement: Supplementary file 3 [file Table2.docx]

**Table S2. The top 10 hub genes and their functions that were selected by the cytoHubba-MCC method.**

| **Rank** | **Gene** | **Full name** | **Function** |
| --- | --- | --- | --- |
| 1 | CCNA2 | cyclin A2 | The protein belongs to the highly conserved cyclin family, whose members function as regulators of the cell cycle. |
| 2 | TOP2A | DNA topoisomerase II alpha | Encodes DNA Topoisomerase, an Enzyme that controls and alters the topologic states of DNA during transcription. Involved in processes such as chromosome condensation, chromatid separation, and the relief of torsional stress that occurs during DNA transcription and replication. |
| 3 | CDK1 | cyclin-dependent kinase 1 | Plays a key role in the control of the eukaryotic cell cycle by modulating the centrosome cycle as well as mitotic onset; promotes G2-M transition via association with multiple interphase cyclins. |
| 4 | ESPL1 | extra spindle pole bodies like 1, separase | Stable cohesion between sister chromatids before anaphase and their timely separation during anaphase are critical for chromosome inheritance. |
| 5 | KIF2C | kinesin family member 2C | Encodes A kinesin-like protein that functions as a microtubule-dependent molecular motor. The encoded protein can depolymerize microtubules at the plus end, thereby promoting mitotic chromosome segregation. Alternative splicing results in multiple transcript variants. |
| 6 | PBK | PDZ binding kinase | A serine/threonine protein kinase related to the dual specific mitogen-activated protein kinase kinase (MAPKK) family. |
| 7 | UHRF1 | ubiquitin-like with PHD and ring finger domains 1 | The protein binds to specific DNA sequences and recruits a histone deacetylase to regulate gene expression.  It is regarded as a hub protein for the integration of epigenetic information. |
| 8 | RACGAP1 | Rac GTPase-activating protein 1 | This protein binds activated forms of Rho GTPases and stimulates GTP hydrolysis, which results in negative regulation of Rho-mediated signals. This protein plays a regulatory role in cytokinesis, cell growth, and differentiation. |
| 9 | PCLAF | PCNA clamp associated factor | Enables chromatin binding activity. Involved in several processes, including cellular macromolecule biosynthetic process; centrosome cycle; and response to UV. |
| 10 | RAD51 | RAD51 recombinase | The protein is a member of the RAD51 protein family, involved in the homologous recombination and repair of DNA. |
